# Evaluation of mucin-1, nuclear factor κB, and hemoglobin A1c levels in obese and non-obese individuals

**DOI:** 10.1590/1806-9282.20231214

**Published:** 2024-05-03

**Authors:** Müjde Fadıloğlu, Ahmet Sarper Bozkurt, Ersin Akarsu, Şenay Görücü Yilmaz, Zeynel Abidin Sayiner, Hasan Ulusal

**Affiliations:** 1 Gaziantep University, Medicine Faculty, Department of Physiology – Gaziantep, Turkey.; 2 Gaziantep University, Medicine Faculty, Department of Endocrinology and Metabolism – Gaziantep, Turkey.; 3 Gaziantep University, Faculty of Health Sciences, Department of Nutrition and Dietetics – Gaziantep, Turkey.; 4 Gaziantep University, Faculty of Medicine, Department of Medical Biochemistry – Gaziantep, Turkey.

**Keywords:** Obesity, MUC1, NF-κB, HbA1c

## Abstract

**OBJECTIVE:**

Obesity is a chronic multisystem disease associated with increased morbidity and mortality. Obesity, which is a complex, multifactorial, and heterogeneous condition, is thought to result from the interaction of environmental, physiological, and genetic factors. In this study, the relationship between serum levels of hemoglobin A1c, mucin-1, and nuclear factor κB in obese and healthy cohorts was evaluated along with biochemical and gene expressions and with demographic and clinical covariates, and their effects on obesity were evaluated.

**METHODS:**

This case–control study included a total of 80 individuals, 40 healthy controls and 40 obesity patients, consisting of female and male aged between 18 and 63 years. Hemoglobin A1c, mucin-1, and nuclear factor κB levels were determined by ELISA in serum samples obtained from patients. In addition, aspartate aminotransferase, alanine transaminase, low density lipoprotein, and glucose values were measured. The gene expressions of the same markers were analyzed by quantitative real-time polymerase chain reaction, and their regulation status was defined.

**RESULTS:**

Serum levels of hemoglobin A1c, mucin-1, and nuclear factor κB were found to be high in obese individuals (p<0.05). The gene expression of these serum markers was found to be upregulated. Of the anthropometric measurements, waist circumference and body mass index were correlated with both serum markers and gene expressions (p<0.05).

**CONCLUSION:**

In addition to the known association of hemoglobin A1c and nuclear factor κB with obesity, serum levels of mucin-1 as well as upregulation of genes point to its modifier effect on obesity. These parameters can be the powerful markers in the diagnosis of obesity.

## INTRODUCTION

Obesity is a serious health problem all over the world. Obesity, which is caused by a decrease in physical activity and the effects of various genetic factors, causes hypertension, dyslipidemia, insulin resistance, and severe psychological stress, and it is seen with an increasing frequency in childhood^1^.

Obesity increases *NF-k*β active in the liver and skeletal muscle and the transcription of *NF-k*β target genes^2^. The*NF-*κ*B* pathway can be activated by cellular stress, inflammatory cytokines, growth factors, or ultraviolet rays^3^. Overfeeding activates the hypothalamic NF-κβ pathway through elevated endoplasmic reticulum stress in the hypothalamus. According to the results obtained here, the hypothalamic *IKKb/NF-*κβ pathway is a general neural mechanism for the energy imbalance underlying obesity^4^. The *MUC1* protein encodes a membrane-bound protein that is a member of the MUC1 family and is also involved in intracellular signaling. This protein is expressed on the apical surface of epithelial cells lining the mucosal surfaces of many different tissues, including lung, breast, stomach, and pancreas^5^. The *MUC1* gene promoter contains potential binding sites for *NF-*κ*B* and similar transcriptional regulatory factors^6^.

Within this transcriptionally important region of the *MUC1* promoter, the putative region for *NF-*κ*B* overlaps with an AP-3 (activator protein 3) element and a STAT (signal converter and activator of transcription)^7^. In many people, increased glucose needs can result in sustained insulin release, constant cravings for food, and eventually obesity^8^.

The data obtained from this study will provide information about the role and diagnostic power of two known and one newly proposed serum markers as well as gene expressions in the formation of obesity in the metabolism of the disease.

## METHODS

### Clinical characteristics of subjects

The study included a total of 80 participants: 40 healthy control individuals (18–62 years old, female=26, male=14) and 40 individuals with obesity (18–63 years old, female=27, male=13). The study group was composed of individuals who applied to Gaziantep University Faculty of Medicine Endocrinology outpatient clinic. Research permission was obtained from Gaziantep University Clinical Research Ethics Committee (Ethical number: 2022/51), and a voluntary consent form was obtained from the participants. The study was supported by Gaziantep University Scientific Research Projects Management Unit (Project number: TF.YLT.22.38). In addition to the family history of all individuals, age, gender, height, weight, body mass index, and waist circumference measurements were taken (exercise status and duration, meal content and amount of consumption, water consumption, fat consumption, etc.)^9^.

### Inclusion and exclusion criteria

The inclusion criterion was to be over 18 years old. Individuals who were considered obese were classified according to the international standards as a result of body mass index (BMI), waist circumference, and skinfold thickness measurements of both patients and controls (https://www.cdc.gov/obesity/basics/adult-defining.html). The diagnosis of concomitant insulin resistance, hypertension, and atherosclerosis was questioned in the patient group and was not included. Among healthy control subjects, individuals with a BMI of <80 for women and <94 for men with a waist circumference were included in the study. Patient exclusion criteria were also valid for control subjects.

### Measurement of protein activity

Some of the sera obtained from all blood samples were used for the measurement of the protein levels, and the other part was used for the determination of aspartate aminotransferase (AST), alanine transaminase (ALT), glucose, and low density lipoprotein (LDL) levels. HbA1c (CEA190Hu, SCN Life Science & Technology Company, Missouri, TX, USA), NF-κB (SEB824Hu, SCN Life Science & Technology Company, Missouri, TX, USA), and MUC1 (SEA413Hu, SCN Life Science & Technology Company, Missouri, TX, USA) serum levels were determined by ELISA reader from the serum samples according to the commercial protocol.

### Quantitative analysis of gene expression

A volume of 3 mL of peripheral blood sample was obtained from all individuals, and RNA isolation was performed by modifying the Trizol method^10^. Quantification of the analyzed total RNA samples was performed with a spectrophotometer. Totally, 20 μl cDNA reaction was prepared, which contained5x RT buffer, dNTP, OneScript^®^Plus RTase, and 2 ng RNA. The samples were incubated at 55°C for 15 min and at 85°C for 5 min in a thermal cycler. Analysis of the expression changes of *HbA1c* [Chromosome 16, NC_000016.10 (176680.177522)], *NF-*κ*B* [Chromosome 4, NC_000004.12 (102501359.102617302)], and *MUC1* [Chromosome 1, NC_000001.11 (155185824.155192915)] genes was carried out by quantitative real-time polymerase chain reaction (qRT-PCR). Normalization of the expressional changes of genes was performed relative to β-actin (*ACTB*) endogenous control. A volume of 25 μl reaction mixture was prepared containing 2X SYBR Green Master Mix, 10X Quanti Tect primer assay, 2 μg cDNA, and ddH_2_O. Samples were incubated in a real-time PCR device for 5 min, 1 cycle at 95°C, 5 s at 95°C, and 40 cycles at 60°C for 10 s. Each sample was replicated three times, and the mean DDCt values were calculated^11^.

### Statistical analysis

Mann-Whitney U test (comparison between two groups) was used to compare the gene expression levels and clinicopathological data. Descriptive statistics of the data obtained from the study are given with mean, standard deviation for numerical variables, and frequency and percentage analysis for categorical variables. The normal distribution test of *HbA1c*, *NF-*κ*B*, and *MUC1* variables was analyzed using the Shapiro-Wilk test. It was determined that the variables other than the *NF-*κ*B* variable did not conform to the normal distribution (p<0.05). Independent samples t-test and Mann-Whitney U test were used to compare these variables according to the study groups. ROC analysis was used to determine the cutoff point for *HbA1c*, *NF-*κ*B*, and *MUC1* variants. All data obtained from patients and laboratory studies were analyzed using the SPSS 22.0 program. p<0.05 was considered significant. The diagnostic power of gene expressions was evaluated by receiver operating characteristic (ROC) curve analysis. The diagnostic power was determined by the area under the curve (AUC) values classification^12^. To detect a difference of 23 units (effect size 23) between the biochemical and expressional measurements of the obesity and control groups, the required minimum sample size was calculated as 40 individuals in each group, under the conditions of 5% Type I error and 80% power (Type II error 0.20). Power analysis was performed with the SPSS 22.0 package program.

## RESULTS

### Clinical and demographic factors

This is a case–control study in randomly selected individuals who were not related but age-sex matched. The cohort consisted of a total of 80 individuals between the ages of 18 and 63 years, with 40 healthy controls and 40 obesity patients.

**Figure 1:**
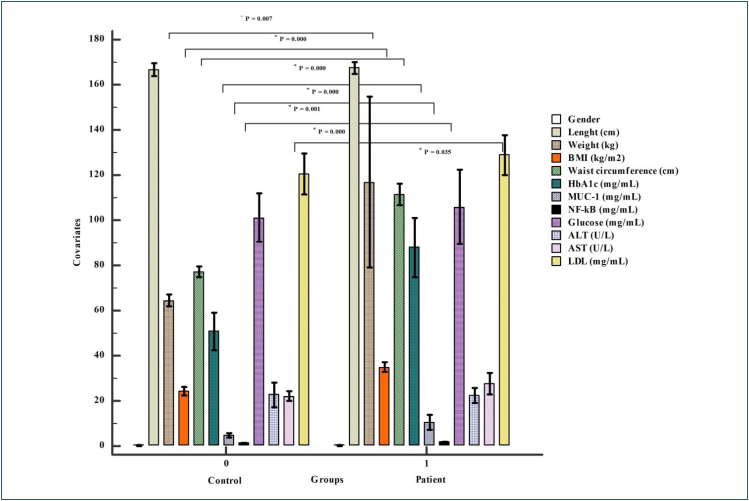
Clustered multiple variable graph. The bar graph represents the 95%CI values for the mean of the covariates in the groups. Weight, BMI, waist circumference, *HbA1c, MUC1, NF-κB*, and AST were statistically significant (p<0.005).

When the average age of the groups was examined, it was determined that the highest percentile was between the ages of 36 and 50 years. It shows that the risk of developing obesity increases with the contribution of covariates in the transition from young to middle age and old age (Figure 1 and Table 1). Descriptive statistics differed significantly between groups for weight, BMI, waist circumference, *HbA1c*, *MUC1*, and *NF-*κ*B*. Weight, waist circumference, and BMI were high in patients as expected. HbA1c, MUC1, and NF-κB serum levels were high, and the significant difference was detected (p<0.05). Glucose levels were not significant. The parameter indicating the absence of diabetes and diabetes onset in individuals indicated the trend with *HbA1c*. ALT and LDL levels were not significant (p>0.05). Elevated AST level may be a sign of many diseases. However, due to the absence of these diseases in the clinical data of our patient group, it is thought to be associated with obesity. Although an increase was observed in LDL levels in the patient group, there was no significant difference (p>0.05). The fact that patients are taking cholesterol medication (n=26) may affect the significance. This suggests the association of obesity with cholesterol in patients. The positive correlation between *HbA1c*, BMI, and waist circumference, which is used in the diagnosis of diabetes and pre-diabetes, indicates that individuals are at risk of developing diabetes. Therefore, in accordance with the literature, it is a marker that can be investigated for diabetes risk in obese individuals. There is also a correlation between *HbA1c*, *MUC1*, and *NF-*κ*B*. In obese individuals, these three parameters with high serum levels seem to be associated with obesity risk in the absence of diabetes.

**Table 1:** Descriptive statistics of cohorts.

Covariates	Groups	Mean	df	SD	SE	95%CI	p-value
Gender	Control	0.35	34	0.483	0.076	0.20–0.50	0.816
	Patient	0.33	33	0.474	0.075	0.17–0.48	
Lenght (cm)	Control	166.65	34	9.404	1.487	163.64–169.66	0.674
	Patient	167.48	33	7.994	1.264	164.92–170.03	
Weight (cm)	Control	64.48	34	7.776	1.229	61.99–66.96	**0.007** *
	Patient	116.88	33	18.669	18.763	78.92–154.83	
BMI (kg/m^2^)	Control	24.09	34	5.94	0.93922	22.190–25.990	**0.000** *
	Patient	34.992	33	6.057	0.9577	33.055–36.930	
Waist circumference (cm)	Control	77.18	34	8.041	1.271	74.60–79.75	**0.000** *
	Patient	111.38	33	14.565	2.303	106.72–116.03	
HbA1c (mg/mL)	Control	50.745	34	50.745	4.13301	42.385–59.104	**0.000** *
	Patient	87.869	33	87.869	6.57524	74.570–101.168	
MUC1 (mg/mL)	Control	4.693	34	4.693	0.50313	3.675–5.710	**0.001** *
	Patient	10.5	33	10.5	1.74677	6.954–14.046	
NF-κB (mg/mL)	Control	1.306	34	1.306	0.5006	1.145–1.466	**0.000** *
	Patient	1.904	33	1.94	0.41727	1.807–2.074	
Glucose (mg/mL)	Control	101.14	34	101.14	31.956	90.48–111.79	0.622
	Patient	105.89	33	105.89	48.853	89.60–122.18	
ALT (U/L)	Control	22.65	34	22.65	16.794	17.05–28.25	0.952
	Patient	22.46	33	22.46	9.714	19.22–25.70	
AST (U/L)	Control	22.11	34	22.11	6.729	19.84–24.39	**0.035** *
	Patient	27.59	33	27.59	13.783	23.00–32.19	
LDL (mg/mL)	Control	120.53	34	120.53	25.773	111.54–129.52	0.181
	Patient	128.84	33	128.84	25.973	120.18–137.50	

* p<0.05 is significant, SD: standard deviation; SE: standard error; CI: confidence interval.

### Gene expression analysis of groups

To clarify the question of whether the direction of the relationship for *HbA1c* and *NF-*κ*B* is due to obesity or obesity caused by these factors, and to show whether *MUC1* has a role in these links, it was determined that all three genes were upregulated in obesity and were statistically significant between groups (p<0.05). *HbA1c*, *NF-*κ*B,* and *MUC1* genes were found to be upregulated and statistically significant in obese individuals (p<0.05) (Table 2).

**Table 2:** Gene expression analysis of groups (according to DDCt values).

Covariates	Groups	Mean	df	SD	SE	95%CI	p-value
*HbA1c*	Control	7.246	1	0.477	0.075	7.093–7.399	**0.001** *
	Patient	13.438	1	0.303	0.048	13.341–13.534	
*MUC1*	Control	6.075	1	0.534	0.084	5.905–6.246	**0.001** *
	Patient	12.752	1	0.495	0.078	12.594–12.911	
*NF-*κ*B*	Control	8.158	1	0.59	0.093	7.964–8.347	**0.001** *
	Patient	12.699	1	1.039	0.164	12.367–13.032	

* p<0.05 is significant, SD: standard deviation; SE: standard error; CI: confidence interval.

In the correlation analyses for the three genes, the expression changes were statistically significant for *HbA1c–MUC1* (Pearson correlation=0.984) and *HbA1c–NF-*κ*B* (Pearson correlation=0.938) (correlation is significant at the 0.01 level) (2-tailed) (p=0.000). In the analysis of gene expressions, the correlation of which was investigated for clinical and demographic data, it was found that they were correlated with all parameters except ALT and were statistically significant. Obtained data show that there is a correlation between gene expressions and BMI, waist circumference, serum *HbA1c*, *MUC1*, *NF-*κ*B* values, and liver enzyme levels in obese individuals.

Upregulations of *HbA1c* and *NF-*κ*B* and high serum levels have been shown to be associated with obesity.

### Diagnostic power of gene expressions and covariates with receiver operating characteristic analysis

In the ROC curve analyses performed to determine the diagnostic power of the datasets, BMI and waist circumference measurements were found to be excellent. *NF-*κ*B*, *HbA1c*, *MUC1*, and AST levels were determined as very good, good, satisfactory, and unsatisfactory, respectively, for serum levels. In multivariate analyses based on the ROC values of the datasets, covariates were modeled together, and their diagnostic power was evaluated. It was determined that there was diagnostic power in binary combinations (except *MUC1* and AST) with *HbA1c*. For *MUC1*, the diagnostic power was statistically significant when evaluated together with *NF-*κ*B*, BMI, waist circumference, and AST.

The diagnostic power of the upregulation of genes in obese individuals was evaluated with the ROC curve. Genes were found to have "excellent" diagnostic power in differentiating obese from healthy individuals.

## DISCUSSION

Obesity is a complex, multifactorial disease involving genetic and environmental factors. There is an increase in the prevalence of obesity worldwide in both developed and developing countries. There are no studies on the reflections of the relationship between *MUC1* and the other two genes (*HbA1c* and *NF-*κ*B*) in obesity physiology. Obesity can affect the level of *HbA1c*. There was a significant correlation between anthropometric measurements and *HbA1c*, with *HbA1c* levels being higher in the obese group, and significant patient scores with HbA1c and high body fat indicate the relationship of the molecule with obesity^13^. Our data also support these findings.

During this process, *MUC1* is thought to be involved in cell growth and division (proliferation), helping cells stick together (cell adhesion), cell movement (motility), and cell survival. Some researchers suggest that in the nucleus, *MUC1-CT* helps control the activity of other genes^14-16^. *MUC1* can also provide information on the direction of the relationship between obesity and cancer. Several possible mechanisms have been proposed to explain how obesity may increase the risks of some cancers^17,18^. Current results have shown that the *MUC1* gene may play a critical role in determining obesity^19^. The data in this study indicate that upregulation of *MUC1* is associated with an increased risk of obesity. *MUC1* is known to regulate the expression of metabolic genes as a transcriptional activator^20^. The role of this mission is great in its relationship with obesity. *MUC1* receptor tyrosine kinase affects the activity and stability of transcription factors by regulating metabolic activities, due to its modulatory role in the signaling of the receptor. The correlation analyses suggest that *NF-*κ*B* may have a role in this task.

Nuclear factor κB pathway, cellular stress, inflammatory cytokines, growth factors, ultraviolet rays, etc. can be activated. Activated *NF-kB* is transported from the cytoplasm to the nucleus and then binds to a specific DNA sequence, forming a DNA–NF-κB complex^3^. Inappropriate activation of *NF-*κ*B* is associated with a number of inflammatory diseases, while persistent inhibition of *NF-*κ*B* leads to inappropriate immune cell development or delayed cell growth^21^.

Receiver operating characteristic analyses are helpful in eliminating the confounding effects of clinical risk factors and environmental exposures. The analyses were carried out to predict the variables and their effects indicate that genes have biomarker potential. These genes are possible targets for detecting disease as they move away from a healthy state. These genes can be used to determine the appropriate option in various clinical conditions with a threshold value, to establish the balance of sensitivity and susceptibility, and to ensure the natural balance that exists between sensitivity and susceptibility.

In conclusion, *MUC1* appears to be capable of modulation on its own, without being overshadowed by *HbA1c* and *NF-*κ*B* in obesity. However, diagnostic tests indicate that there is no strong marker to distinguish patients from healthy ones. For a better understanding of *MUC1* pathology and physiology, studying in large-scale groups will make it possible to use it as a treatment target.

## Data Availability

The datasets generated during and/or analyzed during the current study are available from the corresponding author upon reasonable request.
